# Cell-Based Therapies for Periodontal Regeneration: A Systematic Review and Meta-Analysis with Future Implications for Personalized Medicine

**DOI:** 10.3390/jpm16090469

**Published:** 2026-09-13

**Authors:** Rafael X. Erazo Vaca, Gabriela G. Zambrano Manzaba, Luis-Chauca Bajaña, Andrea-Ordoñez Balladares, Andres-Villao Leon, Nayely J. Teran Sanchez, Gema N. Mendoza Manzaba, Rito A. Salazar Román, Jorge L. Teran Sanchez, David A. Flor Ronquillo, Byron-Velasquez Ron

**Affiliations:** 1 College of Dentistry, University of Guayaquil, Guayaquil 090101, Ecuador; rafael.erazov@ug.edu.ec (R.X.E.V.);; 2 School of Dentistry, Universidad Católica de Santiago de Guayaquil (UCSG), Guayaquil 090101, Ecuador; 3 Facultad de Ciencias de la Salud, Universidad Católica de Santiago de Guayaquil (UCSG)–SOADCO, Guayaquil 090615, Ecuador; 4 College of Dentistry, Universidad Bolivariana del Ecuador, Durán 092406, Ecuador; 5 School of Medicine, Faculty of Medical Sciences, Catholic University of Santiago de Guayaquil, Guayaquil 090615, Ecuador; 6 Unidad de Ciencias Básicas, Escuela Superior Politécnica Agropecuaria de Manabí Manuel Félix López, Calceta 130601, Ecuador; 7 Carrera de Odontología, Department Prosthesis Research, Universidad de Las Américas (UDLA), Quito 170102, Ecuador; byron.velasquez@udla.edu.ec

**Keywords:** mesenchymal stem cells, periodontal regeneration, personalized medicine, precision periodontics, regenerative medicine, periodontitis, periodontal attachment loss

## Abstract

**Background/Objectives**: Periodontitis is a chronic inflammatory disease characterized by progressive destruction of the periodontal supporting tissues. Regenerative responses may vary according to patient-related biological characteristics, periodontal defect morphology, the local inflammatory microenvironment, cellular source, biomaterial selection, and delivery strategy. Cell-based regenerative therapies may therefore provide a biological foundation for the future development of personalized periodontal regenerative approaches. This systematic review and meta-analysis evaluated the effectiveness of cell-based therapies for periodontal regeneration and considered how the current evidence may inform future personalized regenerative strategies. **Methods**: A comprehensive electronic search was conducted in MEDLINE via PubMed, Web of Science, Embase, and the Cochrane Library, with additional searches performed in SciELO and Latindex, in accordance with PRISMA guidelines. Randomized and quasi-randomized controlled clinical trials evaluating cell-based regenerative therapies for periodontal defects were included. The primary outcomes were clinical attachment level (CAL) gain and probing depth (PD) reduction, while radiographic bone regeneration was considered a secondary outcome. Standardized mean differences (SMDs) with 95% confidence intervals (CIs) were calculated using random-effects models. Leave-one-out sensitivity analyses were performed to assess the stability of the pooled estimates. **Results**: Six clinical trials were included in the qualitative synthesis, and five contributed to the quantitative analysis. Cell-based regenerative therapies showed a small, non-significant effect for CAL gain (SMD = 0.17; 95% CI: −0.12 to 0.45; I^2^ = 0%) and PD reduction (SMD = 0.13; 95% CI: −0.22 to 0.48; I^2^ = 33.0%). Radiographic bone regeneration showed a larger favorable effect, although statistical significance was not reached and substantial heterogeneity was present (SMD = 1.25; 95% CI: −0.08 to 2.58; I^2^ = 88.2%). Leave-one-out analyses did not materially alter the non-significant interpretation for CAL or PD, although some variation in effect magnitude and direction was observed. Radiographic bone regeneration was more sensitive to the exclusion of individual studies. Overall, superiority of cell-based regenerative therapies over control interventions was not demonstrated. Risk-of-bias assessment indicated a low overall risk for most randomized trials, with some concerns in one trial and moderate risk in the quasi-randomized study. **Conclusions**: Cell-based regenerative therapies showed preliminary but inconclusive signals of benefit for periodontal regeneration. However, no statistically significant superiority over control interventions was demonstrated for CAL gain, PD reduction, or radiographic bone regeneration. Further well-designed clinical trials are required before these approaches can be considered for routine or personalized periodontal regenerative strategies.

## 1. Introduction

Periodontitis is a chronic inflammatory disease characterized by progressive destruction of the tooth-supporting tissues, including the periodontal ligament, cementum, and alveolar bone, ultimately leading to tooth loss if left untreated [1,2]. Conventional periodontal therapies such as scaling and root planing (SRP), guided tissue regeneration (GTR), and bone grafting procedures are effective in reducing inflammation and controlling disease progression; however, complete and predictable regeneration of the periodontal apparatus remains a major clinical challenge [3,4].

Recent advances in regenerative medicine and tissue engineering have stimulated growing interest in stem cell-based therapies for periodontal regeneration [5,6]. Mesenchymal stem cells (MSCs) possess multilineage differentiation capacity, immunomodulatory properties, angiogenic potential, and the ability to promote tissue repair through paracrine signaling mechanisms [7,8]. Different stem cell populations have been investigated for periodontal regenerative applications, including periodontal ligament stem cells (PDLSCs), bone marrow-derived stem cells (BMSCs), dental pulp stem cells (DPSCs), adipose-derived stem cells (ADSCs), and gingival mesenchymal stem cells (GMSCs) [9,10,11].

Among these cell populations, PDLSCs have received particular attention because of their origin within the periodontal ligament and their ability to differentiate into cementoblast-like, fibroblast-like, and osteoblast-like cells involved in periodontal regeneration [12]. The landmark study entitled “Investigation of multipotent postnatal stem cells from human periodontal ligament” first demonstrated the clonogenicity and regenerative potential of PDLSCs, establishing their relevance for periodontal tissue engineering strategies [13]. Similarly, DPSCs and BMSCs have shown promising regenerative capabilities in both experimental and clinical studies [14,15].

The biological rationale for stem cell-based periodontal regeneration is closely associated with the pathophysiology of periodontitis. Chronic inflammation and microbial dysbiosis induce destruction of periodontal tissues and impair the intrinsic regenerative potential of the periodontium [16]. MSCs may modulate the inflammatory microenvironment through secretion of anti-inflammatory cytokines, extracellular vesicles, and growth factors, thereby promoting tissue healing and regeneration [17,18]. Additionally, stem cells may contribute to angiogenesis, osteogenesis, and periodontal ligament formation within periodontal defects [19].

Several tissue engineering strategies have combined stem cells with scaffolds, biomaterials, growth factors, and barrier membranes to enhance regenerative outcomes [20]. Biomaterials such as collagen scaffolds, xenogeneic bone substitutes, hydroxyapatite matrices, and guided tissue regeneration membranes have been used to improve cell adhesion, proliferation, differentiation, and survival within periodontal defects [20]. Despite encouraging findings from preclinical and clinical studies, the currently available evidence remains heterogeneous due to differences in stem cell sources, biomaterials, defect morphology, follow-up periods, and regenerative protocols.

These sources of clinical and methodological variability are directly relevant to personalized and precision medicine. Periodontal regenerative responses may depend not only on the intervention itself but also on patient-specific biological characteristics, the local inflammatory microenvironment, intrinsic healing capacity, periodontal defect morphology, stem cell source, cell-processing characteristics, scaffold composition, and the method of cell delivery. Consequently, the same stem cell-based regenerative strategy may not provide equivalent outcomes across all patients or periodontal defects. A personalized approach would seek to match the cellular product, biomaterial, and delivery protocol to the biological and anatomical characteristics of each clinical scenario rather than applying a uniform regenerative strategy. However, the available clinical trials have primarily reported average treatment effects at the study-group level and have provided limited information regarding patient stratification, defect-specific responses, cell dose, cell viability, passage number, and standardized cellular characterization. These reporting limitations currently prevent the identification of patient profiles or periodontal defect phenotypes that are most likely to benefit from a specific stem cell source or cell–biomaterial combination. Therefore, synthesizing the available evidence may contribute to personalized periodontal medicine by identifying potential sources of treatment-response variability and defining the variables that should be incorporated into future precision-oriented clinical trials.

Previous systematic reviews have suggested that cell-based regenerative therapies may improve periodontal regenerative outcomes compared with conventional therapies alone; however, uncertainty persists regarding the magnitude and consistency of these effects [5,6]. The available clinical evidence remains limited by differences in cellular sources, regenerative protocols, biomaterials, comparator interventions, and follow-up periods, which complicate direct comparison across studies and limit the strength of current conclusions.

Therefore, the aim of the present systematic review and meta-analysis was to evaluate the effectiveness of cell-based regenerative therapies for periodontal regeneration in human periodontal defects, focusing on clinical attachment level (CAL) gain, probing depth (PD) reduction, and radiographic bone regeneration. In addition, the review sought to discuss the potential implications of the available evidence for the future development of personalized periodontal regenerative strategies, considering differences in patient characteristics, periodontal defects, cellular products, biomaterials, and regenerative approaches.

## 2. Materials and Methods

### 2.1. Protocol and Registration

**Figure 1:**
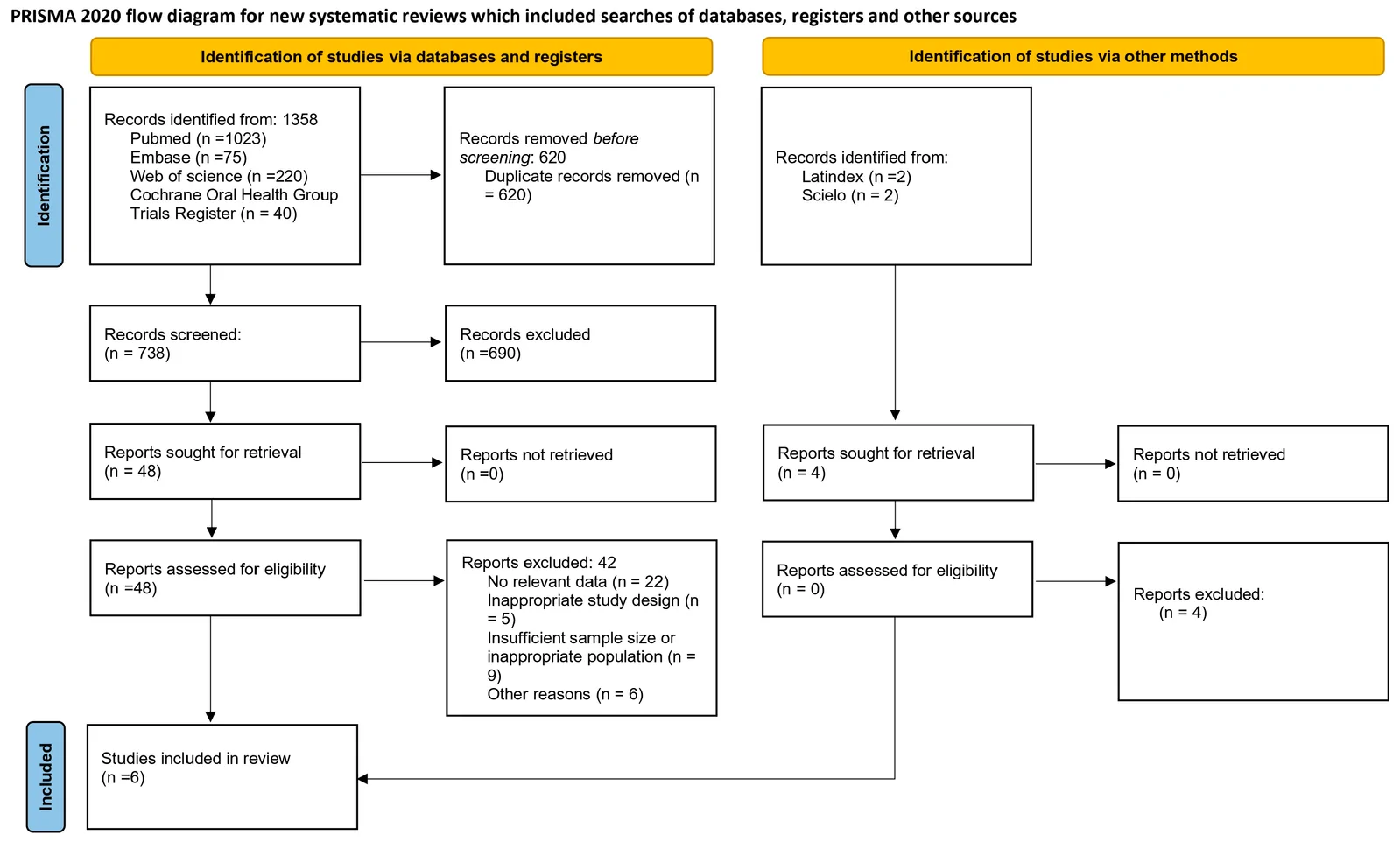
Flowchart of selected studies.

A specific study protocol was developed for the literature search, study selection, data extraction, and statistical analysis processes in accordance with the Preferred Reporting Items for Systematic Reviews and Meta-Analyses (PRISMA) guidelines [21] (Figure 1, File S1). The protocol for this systematic review and meta-analysis on stem cell-based therapies for periodontal regeneration was registered in the International Prospective Register of Systematic Reviews (PROSPERO) under registration number CRD420261395565, aiming to minimize the risk of bias and improve the transparency, methodological rigor, and reliability of the review process.

- Focused Question

The present systematic review and meta-analysis sought to determine whether cell-based regenerative therapies provide additional clinical or radiographic benefits over conventional regenerative approaches in patients with periodontal defects. Accordingly, the PICO framework was defined as follows: P (Population): patients with periodontal defects, particularly intrabony defects associated with periodontitis; I (Intervention): periodontal regenerative approaches involving a viable cellular component with regenerative intent, including periodontal ligament stem cells (PDLSCs), bone marrow-derived mesenchymal stem cells (BMSCs), dental pulp stem cells (DPSCs), adipose-derived stem cells (ADSCs), gingival mesenchymal stem cells (GMSCs), and cultured gingival fibroblast-based preparations, delivered either alone or together with scaffolds, biomaterials, or growth factors; C (Comparator): conventional periodontal regenerative treatment, including scaling and root planing (SRP), guided tissue regeneration (GTR), bone grafting procedures, and cell-free biomaterials; and O (Outcomes): clinical and radiographic measures of periodontal regeneration, with clinical attachment level (CAL) gain and probing depth (PD) reduction considered the primary outcomes, and radiographic bone fill, histological evidence of periodontal regeneration, and safety-related outcomes considered secondary outcomes.

Safety outcomes were also extracted from the included studies, including serious and non-serious adverse events, postoperative complications, treatment-related reactions, tolerability, and adverse healing events when reported. Because the definition and reporting of safety outcomes varied across studies and did not permit meaningful quantitative pooling, safety findings were synthesized descriptively.

### 2.2. Information Sources and Search Strategy

The literature search and study selection process were conducted using the Rayyan QCRI platform (Qatar Computing Research Institute, Doha, Qatar). Following the PRISMA guidelines, a comprehensive electronic search strategy was developed using a combination of Medical Subject Headings (MeSH) terms and free-text keywords related to stem cell-based periodontal regeneration. The main MeSH terms included “Mesenchymal Stem Cells”, “Periodontal Regeneration”, “Periodontitis”, “Periodontal Attachment Loss”, “Alveolar Bone Loss”, and “Guided Tissue Regeneration, Periodontal”. Additional keywords and synonyms such as “periodontal ligament stem cells”, “PDLSCs”, “bone marrow stem cells”, “BMSCs”, “dental pulp stem cells”, “DPSCs”, “adipose-derived stem cells”, “ADSCs”, “gingival mesenchymal stem cells”, “stem cell therapy”, “intrabony defects”, and “bone regeneration” were also incorporated to maximize search sensitivity.

The electronic search was performed in MEDLINE via PubMed, Web of Science, Embase, and the Cochrane Library. Additional searches were conducted in SciELO and Latindex to identify potentially eligible studies not captured through the main electronic databases. No restrictions regarding publication year were applied, and only studies published in English were considered eligible for inclusion. The last search was conducted in May 2026. The complete database-specific search strategies, including the exact search syntax, Boolean operators, field tags, and the number of records retrieved from each source, are provided in Supplementary Table S1.

### 2.3. Eligibility Criteria

All references identified through the electronic databases were independently screened, and full-text articles were manually assessed for eligibility according to the predefined inclusion and exclusion criteria. Studies were included if they met the following criteria: (1) randomized or quasi-randomized controlled clinical trials evaluating cell-based therapies for periodontal regeneration in human patients with periodontal defects, including intrabony defects, periodontal attachment loss, or periodontitis; (2) studies using mesenchymal stem cell-based interventions such as periodontal ligament stem cells (PDLSCs), bone marrow-derived stem cells (BMSCs), dental pulp stem cells (DPSCs), adipose-derived stem cells (ADSCs), gingival mesenchymal stem cells (GMSCs), or other autologous cell-based regenerative therapies with a biological rationale for periodontal tissue regeneration, applied alone or combined with scaffolds, biomaterials, or growth factors; (3) studies including a control or comparison group treated with conventional periodontal therapies, such as scaling and root planing (SRP), guided tissue regeneration (GTR), bone grafts, or cell-free biomaterials; (4) studies reporting at least one clinically relevant periodontal regenerative outcome, including clinical attachment level (CAL) gain, probing depth (PD) reduction, radiographic bone fill, or bone regeneration; (5) studies with a minimum follow-up period of 3 months; and (6) studies published in peer-reviewed journals in the English language.

The exclusion criteria were as follows: (1) in vitro studies and animal or preclinical investigations; (2) case reports, case series, observational studies, reviews, systematic reviews, meta-analyses, editorials, letters, or conference abstracts without the full text; (3) studies not involving stem cell-based therapies for periodontal regeneration; (4) studies using only biomaterials or growth factors without stem cells; (5) studies in which the stem cell source was not clearly specified; (6) studies not reporting relevant clinical periodontal outcomes or lacking sufficient quantitative data for meta-analysis; (7) duplicate publications; and (8) studies with incomplete or unavailable full-text data.

### 2.4. Study Selection and Data Extraction Process

Study selection and data extraction were independently performed by two reviewers (LC and AOB) using a standardized data extraction form specifically developed for this systematic review and meta-analysis. Any disagreements between the reviewers during the screening or extraction process were resolved by a third reviewer (BVR) until consensus was achieved. A formal inter-reviewer agreement coefficient was not prospectively recorded. The following data were extracted from the included studies: first author and year of publication, country, study design, number of participants, total number of periodontal defects, type of control condition, number of defects in the control groups, type of mesenchymal stem cell (MSC)-based intervention, number of defects treated with stem cells, additional regenerative techniques or biomaterials used, number of defects treated with other regenerative approaches, and periodontal regeneration outcomes, including clinical attachment level (CAL) gain, probing depth (PD) reduction, radiographic bone regeneration, histological findings, and safety-related outcomes (Table 1). Variables potentially relevant to personalized regenerative decision-making, including periodontal defect type, cellular source, autologous or allogeneic origin, scaffold or biomaterial characteristics, cell-delivery strategy, adjunctive regenerative procedures, and follow-up duration, were also extracted whenever they were reported. These variables were qualitatively examined to identify factors that may contribute to interindividual and interstudy variability in treatment response. Because patient-level data and reporting of cell-product characteristics were limited, formal patient-stratified analyses could not be performed.

Initially, the titles and abstracts of all retrieved records were screened for potential eligibility. Subsequently, full-text assessment of the selected studies was performed according to the predefined inclusion and exclusion criteria. In cases where essential quantitative or methodological information was unclear or missing, attempts were made to contact the corresponding authors of the studies to obtain additional clarification and ensure the accuracy and completeness of the extracted data.

### 2.5. Risk of Bias Assessment

The methodological quality and risk of bias of the included studies were independently assessed by two reviewers (LC and AOB) according to study design. Randomized clinical trials were evaluated using the Cochrane Risk of Bias 2 (RoB 2) tool [27,28]. Any disagreements between reviewers were resolved through discussion with a third reviewer until consensus was achieved. The following RoB 2 domains were evaluated: bias arising from the randomization process, bias due to deviations from intended interventions, bias due to missing outcome data, bias in outcome measurement, and bias in the selection of the reported results. Each domain was classified as “low risk”, “some concerns”, or “high risk” according to the RoB 2 guidelines.

Because Sánchez et al. (2020) [23] was a quasi-randomized clinical trial, it was assessed separately using the Risk Of Bias In Non-randomized Studies of Interventions (ROBINS-I) tool. The evaluated domains included bias due to confounding, selection of participants, classification of interventions, deviations from intended interventions, missing data, measurement of outcomes, and selection of the reported result. ROBINS-I judgments were classified as low, moderate, serious, or critical risk of bias.

Risk-of-bias judgments were considered in relation to the periodontal outcomes included in the quantitative synthesis, particularly clinical attachment level (CAL) gain, probing depth (PD) reduction, and radiographic bone regeneration. Particular attention was given to randomization or allocation procedures, completeness of outcome data, consistency of outcome measurement, and selective reporting.

### 2.6. Certainty of Evidence

The certainty of evidence for each meta-analyzed outcome was assessed using the Grading of Recommendations Assessment, Development and Evaluation (GRADE) approach. The assessment considered five domains: risk of bias, inconsistency, indirectness, imprecision, and publication bias. Certainty was evaluated separately for clinical attachment level (CAL) gain, probing depth (PD) reduction, and radiographic bone regeneration. The certainty of evidence was classified as high, moderate, low, or very low according to the GRADE framework. Downgrading decisions were based on the methodological limitations of the contributing studies, the magnitude of statistical heterogeneity, the directness of the evidence, the precision of the pooled estimates, and the possibility of publication bias. Because of the small number of studies available for each outcome, publication bias could not be reliably assessed.

### 2.7. Statistical Analysis

#### 2.7.1. Qualitative Analysis

A qualitative synthesis was conducted to summarize the main methodological and clinical characteristics of the studies that met the inclusion criteria. The included randomized clinical trials were systematically reviewed according to the predefined extraction variables (Table 1), including study design, country, participant characteristics, number of periodontal defects, type of control intervention, type of mesenchymal stem cell (MSC)-based therapy, regenerative biomaterials or adjunctive techniques used, follow-up period, and periodontal regeneration outcomes. The studies were categorized according to the type of stem cell-based intervention and comparator therapies, including conventional periodontal treatment, guided tissue regeneration (GTR), bone grafts, and cell-free biomaterials. Clinical and radiographic periodontal regeneration outcomes, including clinical attachment level (CAL) gain, probing depth (PD) reduction, radiographic bone fill, and safety-related findings, were qualitatively assessed across all included studies.

#### 2.7.2. Meta-Analysis

The primary outcomes were clinical attachment level (CAL) gain and probing depth (PD) reduction, whereas radiographic bone regeneration was considered a secondary outcome. For studies reporting more than one follow-up assessment, one time point per outcome was included in the quantitative synthesis to avoid repeated contribution of the same study population. The analyses were based on the changes from baseline reported by the original studies, expressed as CAL gain, PD reduction, or radiographic bone gain/fill, rather than on absolute final periodontal measurements. Means, measures of dispersion, and sample sizes were extracted for the intervention and comparator groups. Only comparisons providing sufficient quantitative information for effect-size estimation were included, and no missing dispersion measures were imputed.

When a study included multiple eligible intervention arms sharing the same comparator, intervention groups representing the same broader cell-based treatment strategy were combined when appropriate to avoid duplication of the control group. In Liu et al. (2025) [22], the single- and double-DPSC groups from the investigator-initiated randomized trial were combined into a single DPSC intervention group and compared with the saline group using the full-analysis-set data reported in Supplementary Table S1. Combined means and dispersion estimates were calculated using standard formulas for pooling independent groups.

The unit of analysis followed that reported in the original trials and corresponded to the participant, treated tooth, or periodontal defect, as applicable. The meta-analysis relied on the aggregate summary data reported by the primary studies. When multiple teeth or periodontal defects could originate from the same participant, no additional clustering adjustment was applied at the meta-analysis level unless such dependence had already been accounted for in the original study analysis, because the information required to reconstruct cluster-adjusted estimates, such as intracluster correlation coefficients and detailed cluster sizes, was not consistently available. Consequently, some residual within-participant correlation may remain in studies reporting outcomes at the tooth or defect level. Each study contributed only one effect estimate per outcome to a given meta-analysis, thereby avoiding duplication of the same study population within the pooled analysis.

Effect sizes were expressed as standardized mean differences (SMDs) with 95% confidence intervals (CIs). Although CAL and PD are conventionally measured in millimeters, SMDs were retained as the primary effect measure because the included studies differed in baseline characteristics, follow-up duration, outcome reporting, and measurement protocols. Standardization therefore provided a common effect-size metric across studies with heterogeneous reporting characteristics. SMDs were interpreted primarily as measures of relative treatment-effect magnitude because they are less directly clinically interpretable than raw mean differences. To improve clinical interpretability, additional random-effects analyses using mean differences (MDs) on the original millimeter scale were performed as sensitivity analyses for CAL gain and PD reduction.

Random-effects models were used because clinical and methodological variability was anticipated among studies, particularly in relation to cellular source, regenerative protocol, scaffold or biomaterial, comparator intervention, delivery approach, and follow-up duration. Pooled estimates were calculated using the inverse-variance method, and between-study variance (τ^2^) was estimated using the restricted maximum likelihood (REML) approach. Statistical heterogeneity was assessed using Cochran’s Q test and the I^2^ statistic, and 95% prediction intervals were reported to illustrate the expected range of effects across comparable future studies. Given the small number of studies contributing to each meta-analysis, Hartung–Knapp adjustments were additionally applied as sensitivity analyses to assess the robustness of statistical inference and confidence intervals.

The different cell-based interventions were pooled because they addressed the same broader clinical question: whether the addition of a viable cellular regenerative component provides an additional periodontal regenerative benefit compared with conventional or cell-free regenerative therapy. Nevertheless, the included cellular products, scaffolds, delivery procedures, comparators, and follow-up periods were not considered therapeutically equivalent. Accordingly, pooled estimates were interpreted as average effects across clinically heterogeneous cell-based regenerative strategies rather than as evidence of equivalence among individual interventions.

Forest plots were used to display individual and pooled effect estimates, and funnel plots were inspected qualitatively for possible small-study effects. Leave-one-out sensitivity analyses were performed to determine whether individual studies disproportionately influenced the pooled estimates. Additional sensitivity analyses included pooling CAL gain and PD reduction as mean differences on the original millimeter scale and application of the Hartung–Knapp adjustment to the CAL, PD, and radiographic bone-regeneration meta-analyses. Statistical analyses were performed in the R statistical environment using the meta, metasens, and metafor packages through RStudio version 2023.09.1+494.

## 3. Results

### 3.1. Study Selection

The database search identified 1358 records. After removal of 620 duplicate records, 738 records underwent title and abstract screening. Of these, 690 were excluded, leaving 48 reports for full-text assessment. Following full-text assessment, 42 reports were excluded, resulting in six studies being included in the qualitative synthesis. Four additional records were identified through SciELO and Latindex; all were retrieved and assessed, but none met the eligibility criteria (Figure 1).

**Figure 2:**
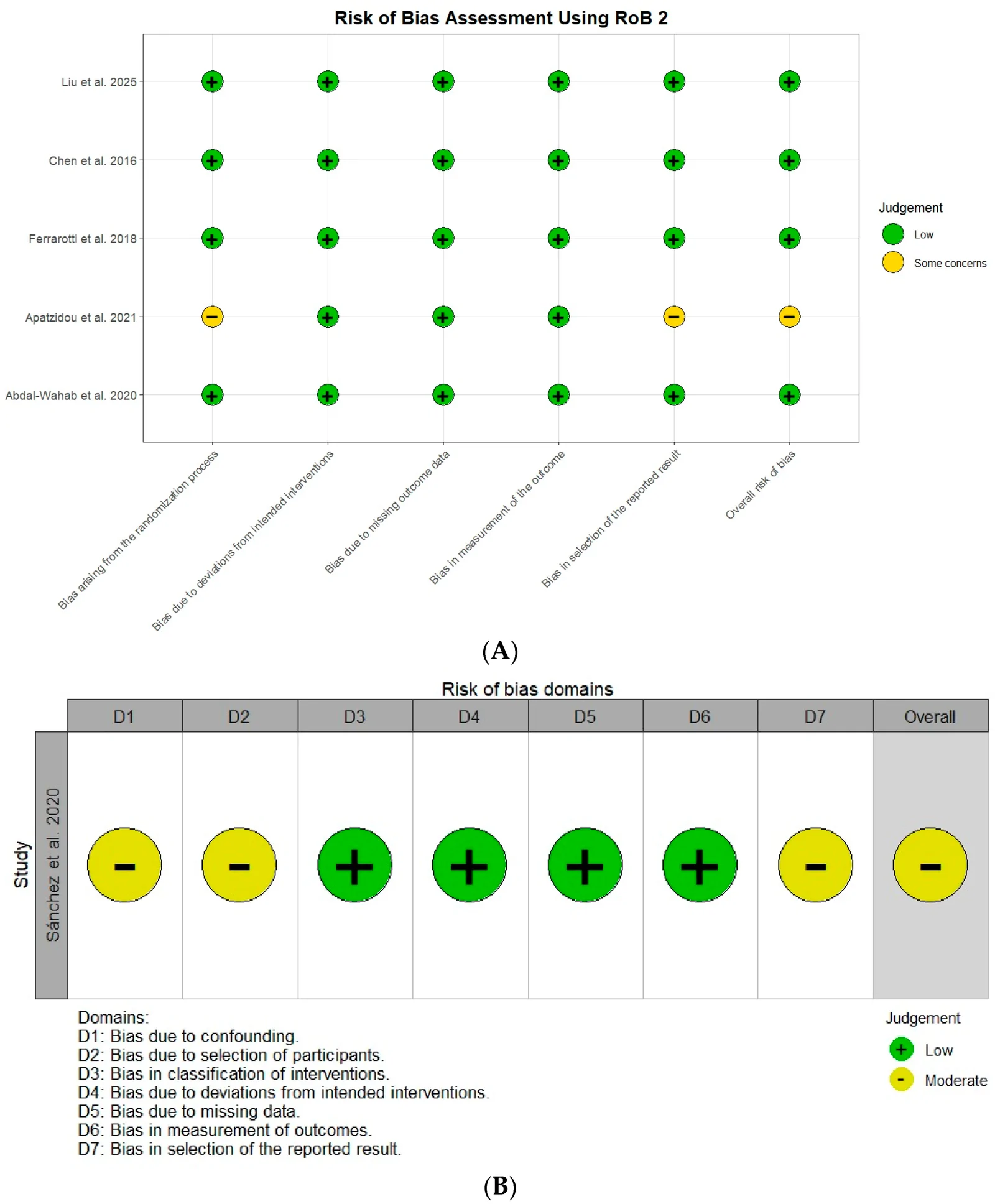
(**A**). Risk-of-bias assessment of randomized clinical trials using the Cochrane Risk of Bias 2 (RoB 2) tool. (**B**). Risk-of-bias assessment of the quasi-randomized trial by Sánchez et al. (2020) [23] using the ROBINS-I tool. Clinical Attachment Level (CAL) [22,23,24,25,26,27].

The risk-of-bias assessment was conducted according to study design. Five randomized clinical trials were evaluated using the RoB 2 tool. Liu et al. (2025) [22], Chen et al. (2016) [25], Ferrarotti et al. (2018) [24], and Abdal-Wahab et al. (2020) [27] were judged to have a low overall risk of bias, whereas Apatzidou et al. (2021) [26] was judged as having some concerns, primarily related to the randomization process and selection of the reported result (Figure 2A). The quasi-randomized trial by Sánchez et al. (2020) [23] was assessed separately using ROBINS-I and was judged to have an overall moderate risk of bias, with concerns mainly related to confounding, selection of participants, and selection of the reported result (Figure 2B). No randomized trial was classified as having a high overall risk of bias, and the quasi-randomized study was not judged to have serious or critical risk of bias.

**Figure 3:**
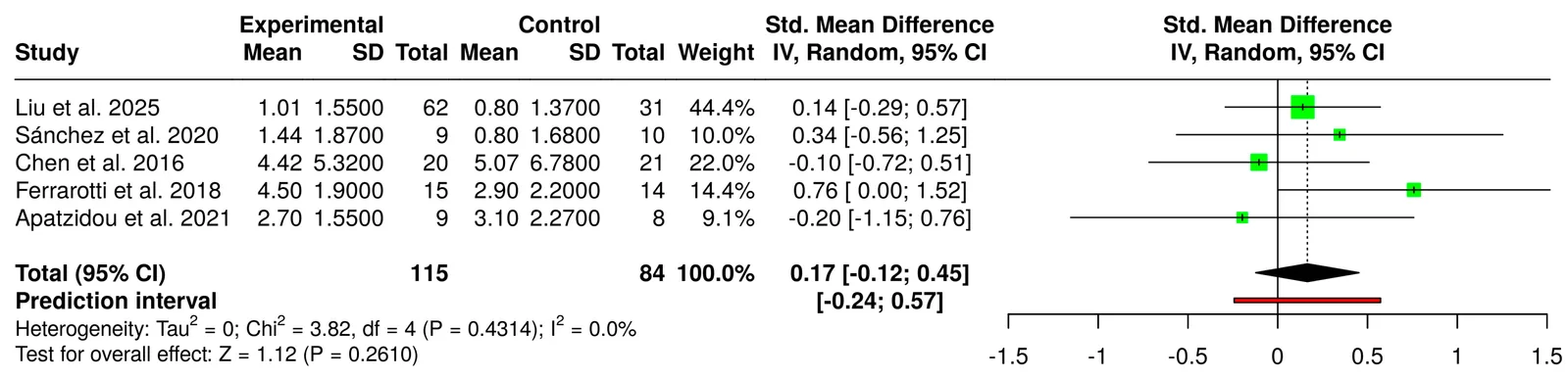

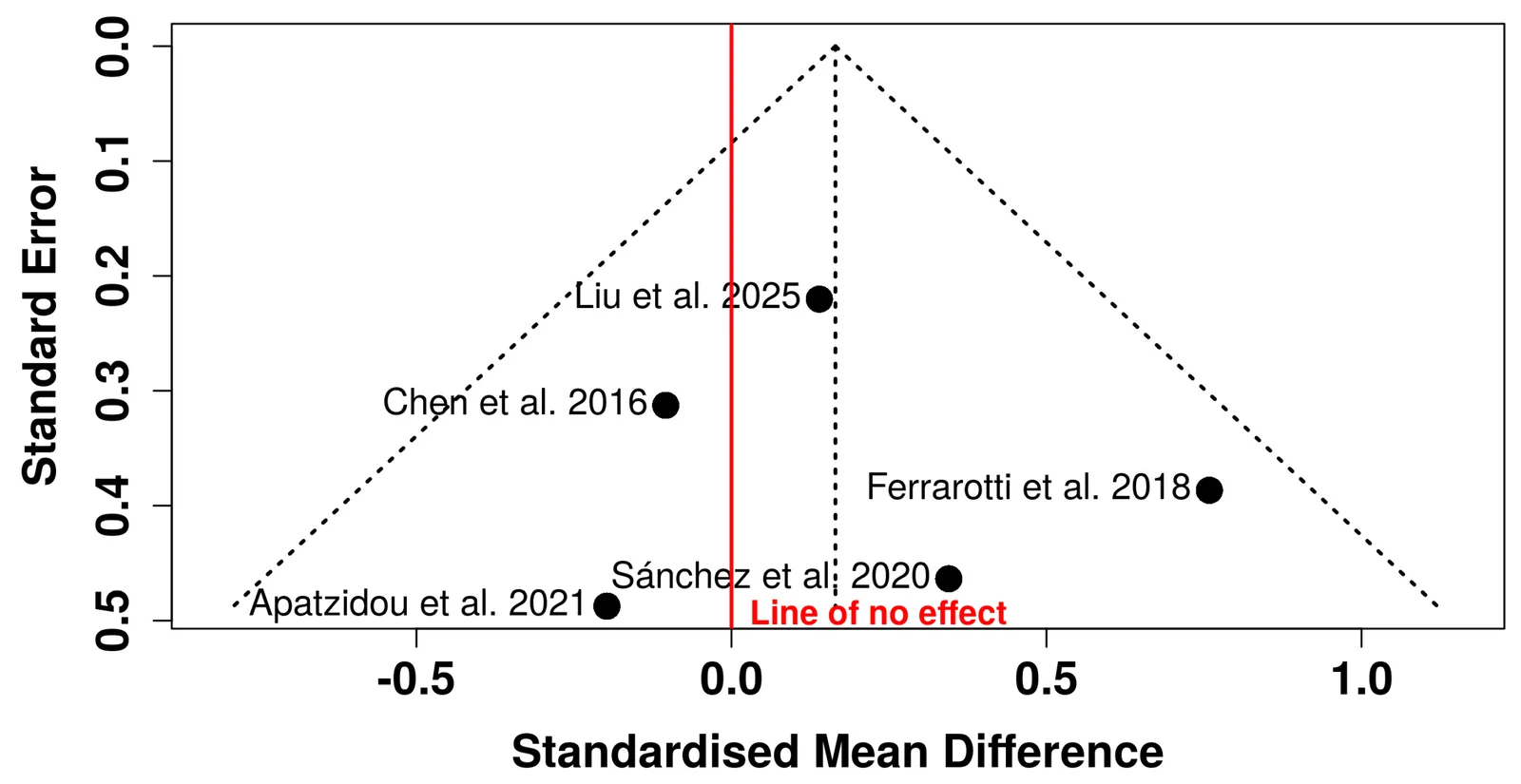
Forest plot and funnel plot of the meta-analysis evaluating clinical attachment level (CAL) gain following cell-based periodontal regenerative therapies [22,23,24,25,26].

Five randomized or quasi-randomized controlled clinical trials evaluating clinical attachment level (CAL) gain following cell-based periodontal regenerative therapies were included in the meta-analysis. The pooled analysis showed a small effect favoring cell-based interventions over control therapies; however, the overall effect was not statistically significant (SMD = 0.17; 95% CI: −0.12 to 0.45; Z = 1.12; *p* = 0.2610). Ferrarotti et al. (2018) [24] showed the largest favorable effect, whereas Liu et al. (2025) [22] and Sánchez et al. (2020) [23] showed smaller positive effects. Chen et al. (2016) [25] and Apatzidou et al. (2021) [26] showed effects close to the null or favoring the control group. No between-study heterogeneity was observed (I^2^ = 0.0%; τ^2^ = 0), and the 95% prediction interval ranged from −0.24 to 0.57. Visual inspection of the funnel plot did not reveal a clear pattern of marked asymmetry; however, this assessment should be interpreted cautiously because only five studies were available (Figure 3).

**Figure 4:**
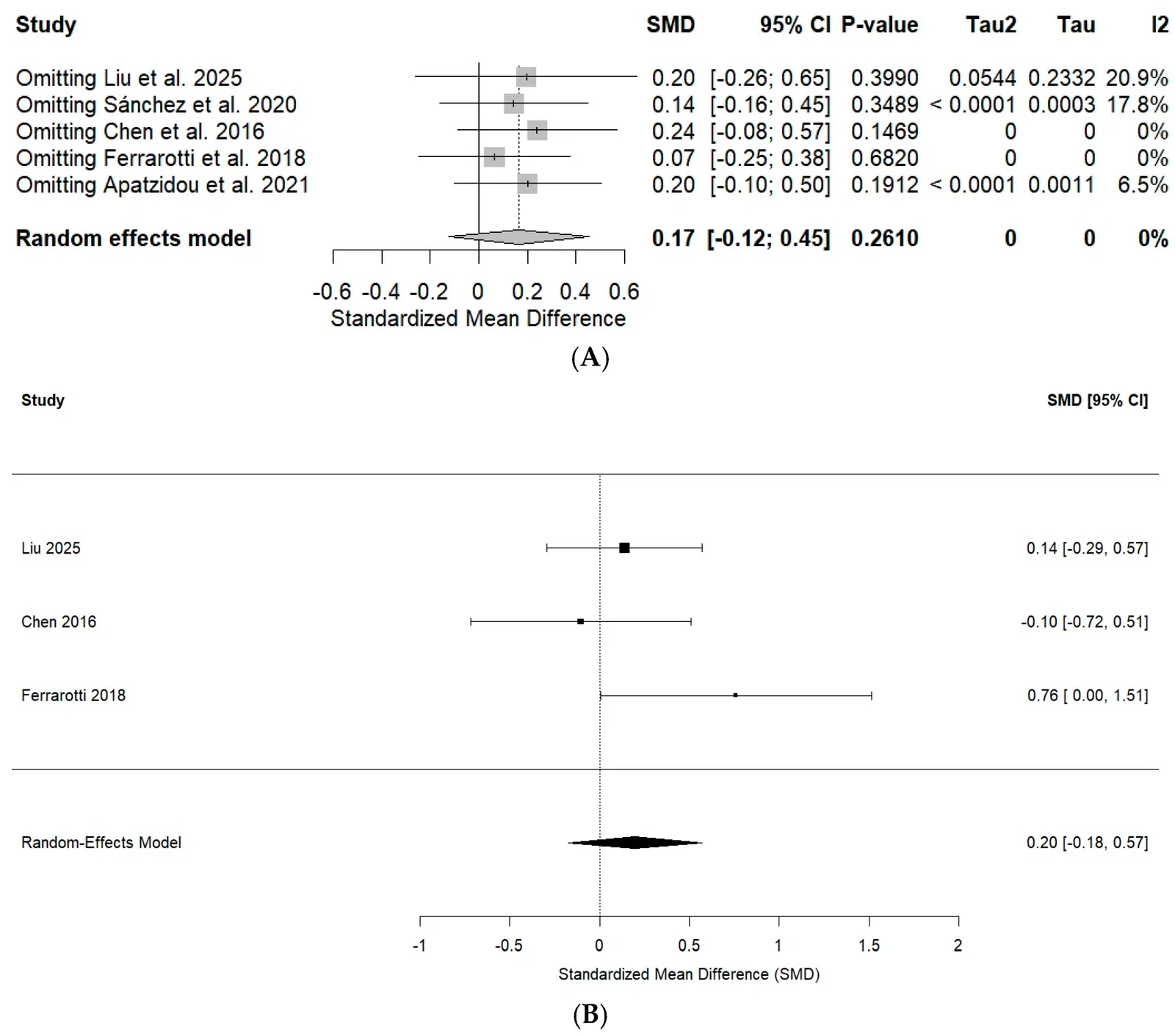
Sensitivity analyses for clinical attachment level (CAL) gain following cell-based periodontal regenerative therapies. (**A**) Leave-one-out sensitivity analysis. (**B**) Sensitivity analysis restricted to studies with a low overall risk of bias [22,23,24,25,26].

The leave-one-out sensitivity analysis demonstrated that the pooled effect for clinical attachment level (CAL) gain remained statistically non-significant after sequential exclusion of each individual study. The pooled estimates ranged from an SMD of 0.07 (95% CI: −0.25 to 0.38) after exclusion of Ferrarotti et al. (2018) [24] to 0.24 (95% CI: −0.08 to 0.57) after exclusion of Chen et al. (2016) [25]. Exclusion of Liu et al. (2025) [22] yielded an SMD of 0.20 (95% CI: −0.26 to 0.65). None of the leave-one-out analyses reached statistical significance, and heterogeneity remained low (I^2^ = 0–20.9%) (Figure 4A). In addition, a sensitivity analysis restricted to studies with a low overall risk of bias yielded an SMD of 0.20 (95% CI: −0.18 to 0.57), maintaining the non-significant interpretation of the primary CAL analysis (Figure 4B).

### 3.2. Probing Depth (PD)

**Figure 5:**
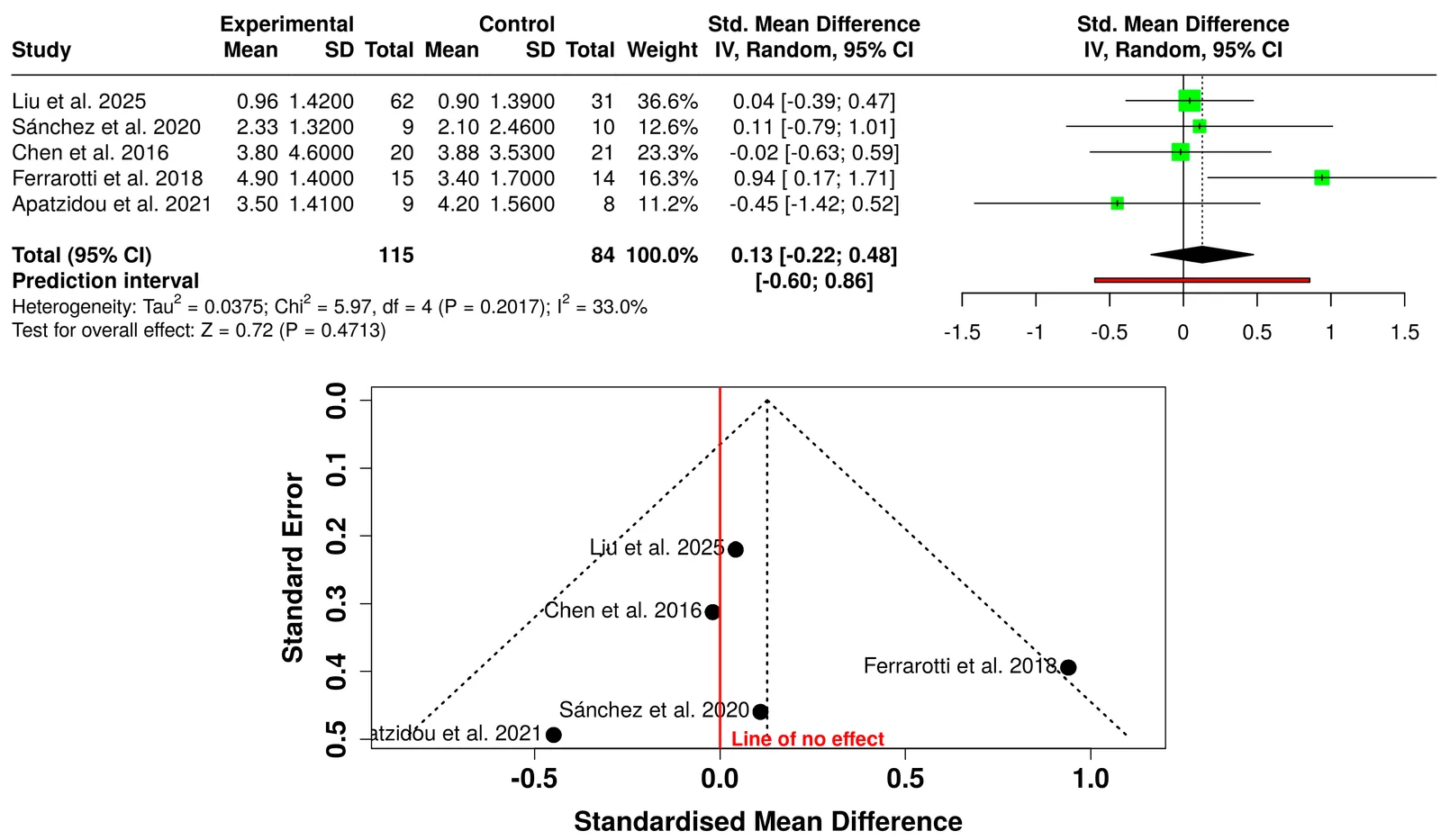
Forest plot and funnel plot of the meta-analysis evaluating probing depth (PD) reduction following cell-based periodontal regenerative therapies [22,23,24,25,26].

Five randomized or quasi-randomized controlled clinical trials assessing probing depth (PD) reduction following cell-based periodontal regenerative therapies were included in the meta-analysis. The pooled analysis showed a small effect favoring cell-based interventions over control therapies; however, the overall effect was not statistically significant (SMD = 0.13; 95% CI: −0.22 to 0.48; Z = 0.72; *p* = 0.4713). Ferrarotti et al. (2018) [24] showed the largest favorable effect, whereas Liu et al. (2025) [22] and Sánchez et al. (2020) [23] showed effects close to the null. Chen et al. (2016) [25] also showed a negligible between-group difference, while Apatzidou et al. (2021) [26] favored the control intervention. Low-to-moderate heterogeneity was observed among studies (I^2^ = 33.0%; τ^2^ = 0.0375; Cochran’s Q = 5.97, *p* = 0.2017). The 95% prediction interval ranged from −0.60 to 0.86, indicating considerable uncertainty regarding the effect expected in a future comparable study. Visual inspection of the funnel plot did not reveal a clear pattern of marked asymmetry; however, this assessment should be interpreted cautiously because only five studies were available (Figure 5).

**Figure 6:**
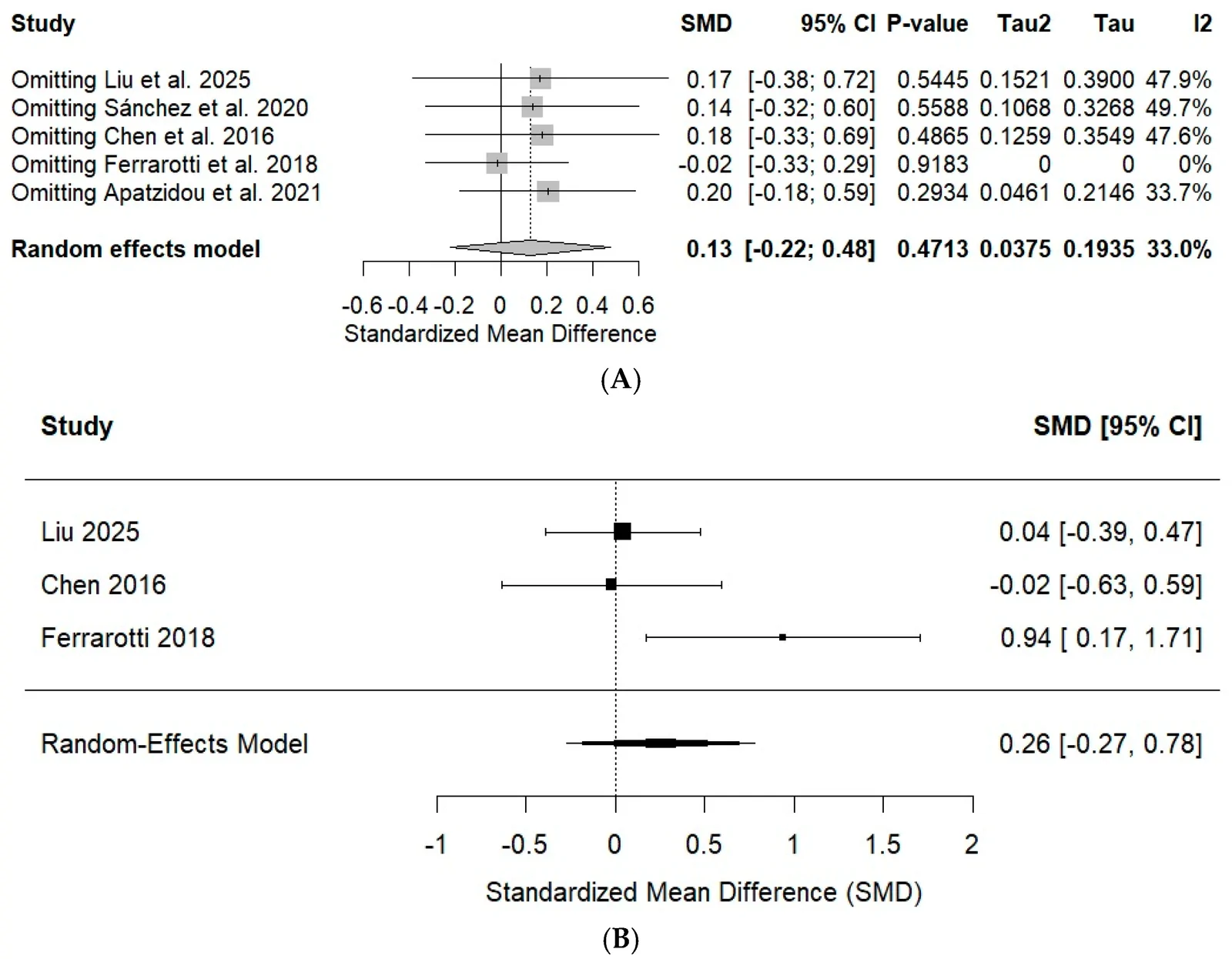
Sensitivity analyses for probing depth (PD) reduction following cell-based periodontal regenerative therapies. (**A**) Leave-one-out sensitivity analysis. (**B**) Sensitivity analysis restricted to studies with a low overall risk of bias [22,23,24,25,26].

The leave-one-out sensitivity analysis showed that the pooled effect for probing depth (PD) reduction remained statistically non-significant after sequential exclusion of each individual study. The pooled estimates ranged from an SMD of −0.02 (95% CI: −0.33 to 0.29) after exclusion of Ferrarotti et al. (2018) [24] to 0.20 (95% CI: −0.18 to 0.59) after exclusion of Apatzidou et al. (2021) [26]. Exclusion of Liu et al. (2025) [22] resulted in an SMD of 0.17 (95% CI: −0.38 to 0.72), indicating that the overall statistical interpretation was not dependent on this study. None of the leave-one-out analyses reached statistical significance. Heterogeneity ranged from 0% after exclusion of Ferrarotti et al. (2018) [24] to approximately 50% after exclusion of Sánchez et al. (2020) [23], suggesting that some variability in the pooled estimates was attributable to individual studies (Figure 6A). In addition, a sensitivity analysis restricted to studies with a low overall risk of bias yielded an SMD of 0.26 (95% CI: −0.27 to 0.78), maintaining the non-significant interpretation of the primary PD analysis (Figure 6B).

### 3.3. Radiographic Bone Regeneration/Bone Fill

**Figure 7:**
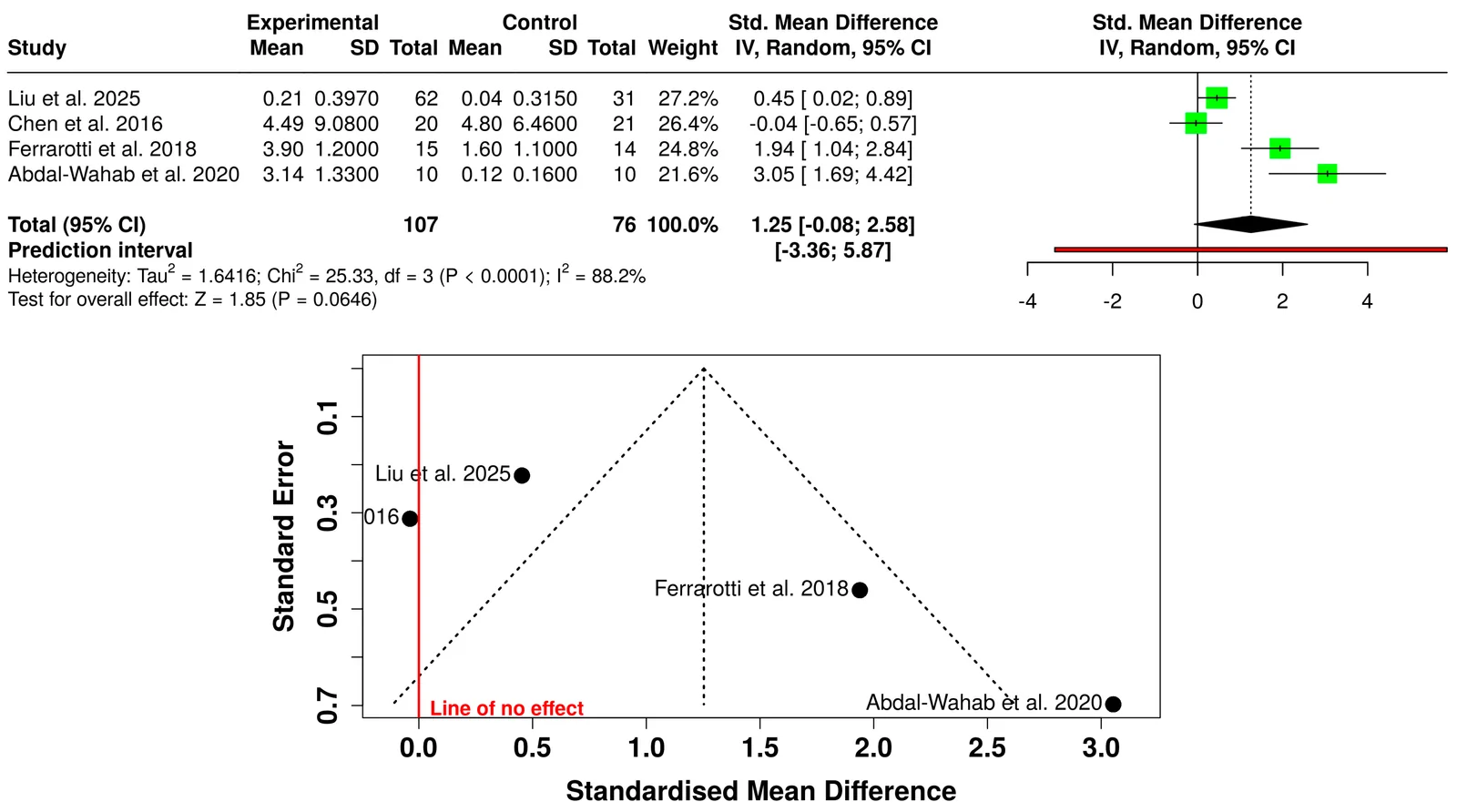
Forest plot and funnel plot of the meta-analysis evaluating radiographic bone regeneration following cell-based periodontal regenerative therapies [22,24,25,27].

Four controlled clinical trials evaluating radiographic bone regeneration or bone fill following cell-based periodontal regenerative therapies were included in the meta-analysis. The pooled analysis showed a large effect favoring cell-based regenerative interventions; however, the overall effect did not reach statistical significance (SMD = 1.25; 95% CI: −0.08 to 2.58; Z = 1.85; *p* = 0.0646). Abdal-Wahab et al. (2020) [27] and Ferrarotti et al. (2018) [24] showed the largest favorable effects, while Liu et al. (2025) [22] demonstrated a smaller positive effect and Chen et al. (2016) [25] showed an effect close to the null. Substantial between-study heterogeneity was observed (I^2^ = 88.2%; τ^2^ = 1.6416; Cochran’s Q = 25.33, *p* < 0.0001), indicating considerable variability in treatment effects across studies. The 95% prediction interval ranged from −3.36 to 5.87, indicating substantial uncertainty regarding the effect expected in a future comparable study. Visual inspection of the funnel plot suggested asymmetry; however, this finding should be interpreted with considerable caution because only four studies were available and substantial between-study heterogeneity was present (Figure 7).

**Figure 8:**
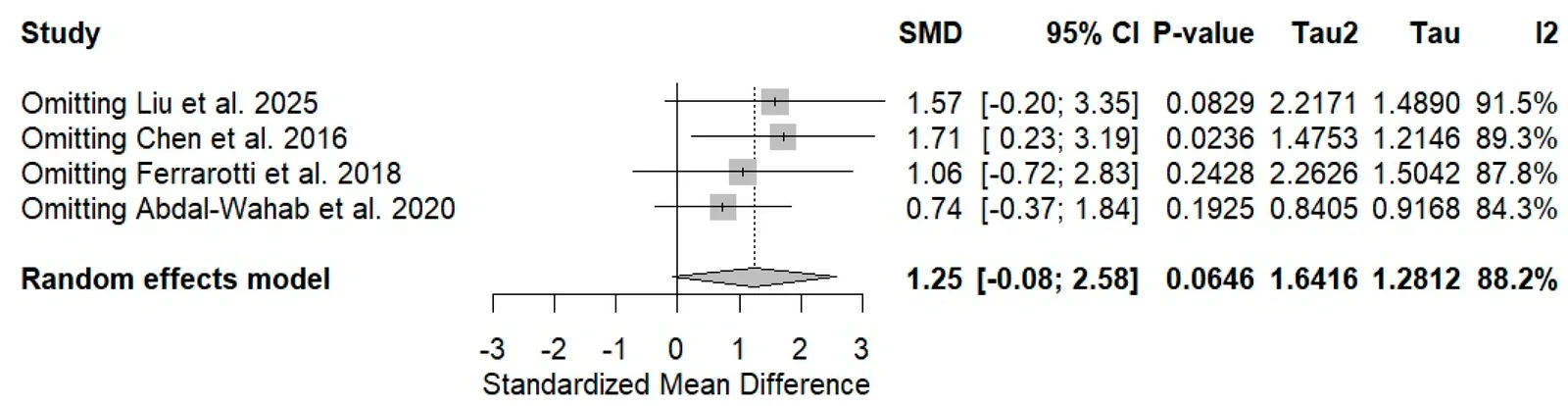
Leave-one-out sensitivity analysis for radiographic bone regeneration following cell-based periodontal regenerative therapies [22,24,25,27].

The leave-one-out sensitivity analysis showed substantial variation in the magnitude of the pooled effect for radiographic bone regeneration. Effect estimates ranged from an SMD of 0.74 (95% CI: −0.37 to 1.84) after exclusion of Abdal-Wahab et al. (2020) [27] to 1.71 (95% CI: 0.23 to 3.19) after exclusion of Chen et al. (2016) [25]. Exclusion of Liu et al. (2025) [22] yielded an SMD of 1.57 (95% CI: −0.20 to 3.35), whereas exclusion of Ferrarotti et al. (2018) [24] resulted in an SMD of 1.06 (95% CI: −0.72 to 2.83). Notably, statistical significance was reached only after exclusion of Chen et al. (2016) [25]. Overall, the sensitivity analysis indicated that the direction of effect generally favored cell-based regenerative therapies, but the magnitude and statistical significance of the pooled estimate were influenced by individual studies, supporting cautious interpretation of this outcome (Figure 8).

### 3.4. Additional Sensitivity Analyses

**Figure 9:**
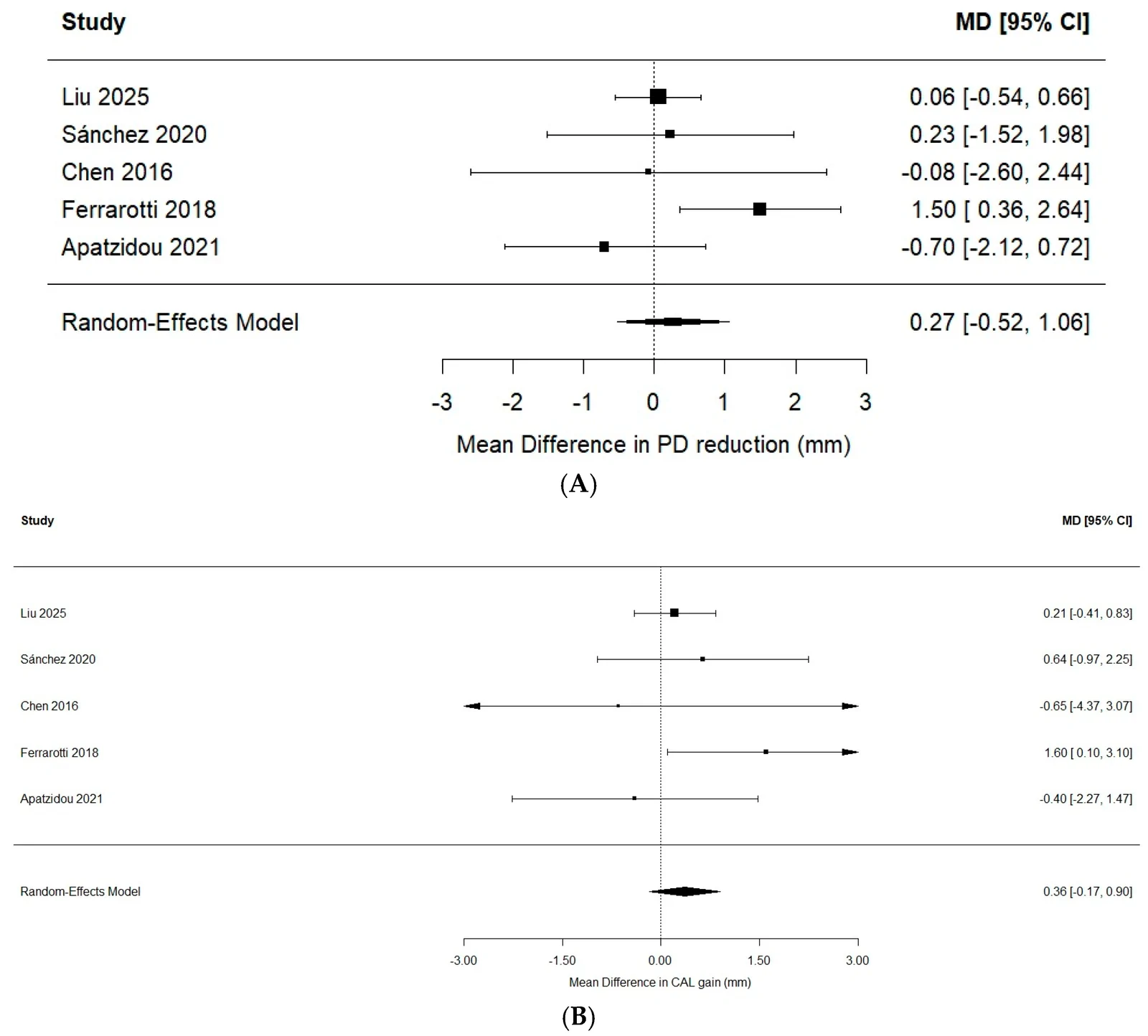
Sensitivity analyses using mean differences on the original millimeter scale. (**A**) Clinical attachment level (CAL) gain. (**B**) Probing depth (PD) reduction [22,23,24,25,26].

Additional sensitivity analyses using mean differences (MDs) on the original millimeter scale were performed to improve the clinical interpretability of CAL gain and PD reduction. For CAL gain, the pooled effect showed a small but statistically non-significant difference favoring cell-based therapies (MD = 0.36 mm; 95% CI: −0.17 to 0.90; *p* = 0.185), with negligible between-study heterogeneity (I^2^ = 2.6%). Ferrarotti et al. (2018) [24] showed a statistically significant individual effect (MD = 1.60 mm; 95% CI: 0.10 to 3.10), whereas the remaining studies showed non-significant effects (Figure 9A). For PD reduction, the pooled mean difference was also not statistically significant (MD = 0.27 mm; 95% CI: −0.52 to 1.06), indicating no clear overall benefit of cell-based therapies over control treatments on the original millimeter scale (Figure 9B). In addition, application of the Hartung–Knapp adjustment to the PD meta-analysis yielded a similarly non-significant effect (SMD = 0.13; 95% CI: −0.42 to 0.68; *p* = 0.546; I^2^ = 25.7%). Overall, the additional sensitivity analyses were consistent with the primary SMD-based findings and did not alter the interpretation of the CAL or PD outcomes.

Application of the Hartung–Knapp adjustment confirmed the robustness of the findings for both CAL gain and radiographic bone regeneration. For CAL gain, the adjusted pooled effect remained non-significant (SMD = 0.17; 95% CI: −0.23 to 0.56; *p* = 0.313), with no observed heterogeneity (I^2^ = 0%). Similarly, for radiographic bone regeneration, the Hartung–Knapp adjustment yielded a non-significant pooled effect (SMD = 1.27; 95% CI: −0.94 to 3.47; *p* = 0.165), with a substantially wider confidence interval, reinforcing the uncertainty surrounding this outcome and supporting a cautious interpretation of the evidence.

### 3.5. Safety and Adverse Events

Safety outcomes were inconsistently reported across the included studies and were therefore synthesized descriptively. Liu et al. (2025) [22] reported no serious adverse events among the 132 participants included across the two randomized trials. In the investigator-initiated trial, two treatment-related grade 1 events occurred in the DPSC groups (toothache and injection-site swelling), while one injection-site swelling event occurred in the saline group; all resolved spontaneously without intervention. Sánchez et al. (2020) [23] reported no serious adverse events and described low postoperative morbidity and appropriate healing. Chen et al. (2016) [25] reported no clinical safety problems attributable to PDLSC transplantation; postoperative findings were mainly limited to moderate swelling and pain, which did not require additional treatment. Apatzidou et al. (2021) [26] reported no adverse healing events during the 12-month follow-up. Ferrarotti et al. (2018) [24] reported no adverse events associated with DPSC treatment. In Abdal-Wahab et al. (2020) [27], a dedicated adverse-event analysis was not clearly reported. Overall, the available studies did not identify serious safety concerns attributable to the evaluated cell-based interventions; however, the limited sample sizes and inconsistent reporting of adverse events preclude definitive conclusions regarding safety.

### 3.6. Certainty of Evidence

The certainty of evidence was rated as low for clinical attachment level (CAL) gain, probing depth (PD) reduction, and radiographic bone regeneration. For CAL and PD, the evidence was downgraded because of risk-of-bias concerns and imprecision, whereas inconsistency and indirectness were not considered serious. For radiographic bone regeneration, the evidence was downgraded because of serious inconsistency and imprecision, whereas risk of bias and indirectness were not considered serious. Indirectness was not downgraded because the included studies directly addressed the review question by evaluating cell-based regenerative interventions in patients with periodontal defects and reporting the prespecified clinical or radiographic periodontal outcomes. Nevertheless, the considerable diversity in cell sources, scaffolds, comparators, delivery protocols, defect characteristics, follow-up periods, and outcome-assessment methods limits the applicability of the pooled estimates to any specific regenerative strategy. Publication bias could not be reliably assessed because fewer than 10 studies contributed to each meta-analysis; therefore, the funnel plots were considered exploratory only, and no inference regarding the presence or absence of publication bias was made. The domain-specific GRADE judgments and reasons for downgrading are summarized in Table 2.

No serious indirectness was identified because the included studies directly evaluated cell-based regenerative interventions in patients with periodontal defects and reported the prespecified clinical or radiographic periodontal outcomes. However, substantial diversity in cell sources, scaffolds, comparators, delivery protocols, defect characteristics, and outcome-assessment methods limits the applicability of the pooled estimates to any specific regenerative strategy.

## 4. Discussion

The present systematic review and meta-analysis suggests that mesenchymal stem cell-based periodontal regenerative therapies may represent a promising therapeutic alternative for enhancing periodontal repair; however, the currently available clinical evidence remains insufficient to confirm a consistent superiority over conventional regenerative therapies. Although the observed effect sizes for clinical attachment level (CAL) gain and probing depth (PD) reduction demonstrated favorable trends toward cell-based therapies, these differences did not reach statistical significance. Radiographic bone regeneration, in turn, exhibited a larger pooled effect estimate, although accompanied by substantial heterogeneity and wide confidence intervals [28,29,30,31]. These findings suggest that the biological impact of stem cell therapies may initially manifest more prominently in processes associated with bone neoformation and tissue remodeling. Nevertheless, the clinical translation toward functional and stable periodontal regeneration is likely dependent on multiple local and systemic variables, including defect morphology, flap stability, inflammatory control, and patient-specific biological characteristics.

Our findings should also be interpreted in relation to previous evidence syntheses. Sun et al. [29] reported statistically significant benefits of stem cell-based therapies for CAL, PPD, and radiographic bone outcomes. In contrast, our updated analysis did not demonstrate statistically significant superiority for CAL gain or PD reduction, although the direction of effect generally favored cell-based therapies. These differences may reflect variations in study eligibility, included cellular interventions, and analytical approaches. Gao et al. [30] similarly highlighted the regenerative potential of mesenchymal stem/stromal cells but emphasized the lack of standardized and predictable clinical protocols. This is consistent with our conclusion that the current evidence remains insufficient to support definitive clinical recommendations.

The absence of statistical significance does not necessarily imply absence of therapeutic effect [31]. This aspect is particularly relevant in periodontal regenerative medicine, where most currently available clinical trials still correspond to pilot studies with small sample sizes and considerable methodological heterogeneity. Furthermore, sensitivity analyses showed that the CAL findings remained stable following sequential exclusion of individual studies, whereas the magnitude of the PD and radiographic bone regeneration estimates showed greater sensitivity to individual studies.

From a biological perspective, the obtained results are consistent with the previously described behavior of mesenchymal stem cells within chronic inflammatory environments. Periodontitis constitutes a complex osteoimmunological condition characterized by progressive destruction of periodontal tissues, disruption of tissue homeostasis, and persistent inflammatory dysregulation [16]. Within this context, MSCs may contribute to periodontal regeneration through immunomodulatory, angiogenic, and trophic mechanisms predominantly mediated by paracrine signaling [17,18]. Growing evidence suggests that a substantial proportion of their therapeutic potential may depend more on their capacity to modulate the inflammatory periodontal niche than on their direct terminal differentiation into mature periodontal tissues. The heterogeneity observed among the included studies likely reflects important biological and methodological differences among the regenerative protocols employed. Factors such as cell source, cellular viability after ex vivo expansion, biomaterial selection, baseline intrabony defect morphology, and follow-up duration may considerably influence the observed clinical response [13,19].

Additionally, several included studies employed advanced regenerative approaches in both experimental and control groups, potentially reducing the ability to detect the incremental benefit specifically attributable to the cellular component [23,25]. This may explain why certain trials demonstrated only modest clinical differences despite exhibiting biologically favorable trends. The absence of statistically significant improvements in CAL and PD may also be partially associated with relatively short follow-up periods in some studies, considering that periodontal tissue maturation and connective tissue remodeling may continue for extended periods following regenerative procedures.

Radiographic bone regeneration was the outcome demonstrating the largest pooled effect estimate, although accompanied by considerable heterogeneity (I^2^ = 88.2%). This finding should be interpreted cautiously. While the radiographic bone neoformation observed may reflect a genuine osteogenic effect associated with stem cell-based therapies, it may also be influenced by differences in imaging methodologies, anatomical variability of periodontal defects, and differences in evaluation periods [3]. Moreover, true periodontal regeneration involves not only bone formation, but also cementum neoformation and functional reorganization of the periodontal ligament. Therefore, radiographic improvement does not necessarily correspond to complete periodontal regeneration from a histological and functional perspective.

Another relevant aspect concerns the role of scaffolds and biomaterials used as cellular delivery platforms. Stem cells require a favorable three-dimensional microenvironment capable of supporting cellular survival, adequate vascularization, and functional tissue organization. Collagen matrices, guided tissue regeneration membranes, and osteoconductive biomaterials may act not only as physical supports, but also as biological modulators of the healing process [20,32]. However, this same therapeutic diversity represents an important source of clinical and methodological heterogeneity, complicating direct comparisons among regenerative protocols and limiting the possibility of establishing standardized therapeutic recommendations.

From a clinical perspective, cell-based therapies may represent a particularly attractive alternative for complex periodontal defects with limited regenerative predictability using conventional approaches. Nevertheless, their routine incorporation into clinical practice still faces important challenges related to biomedical regulation, cell processing, operational costs, reproducibility, and therapeutic standardization [33]. Furthermore, periodontal regeneration remains highly dependent on multiple clinical variables, including plaque control, smoking status, flap stability, and long-term periodontal maintenance, all of which may substantially modify the clinical response independently of the biological potential of the cellular product utilized.

Although the overall methodological quality of the included studies was considered acceptable and no trial was classified as presenting high risk of bias, several limitations related to sample size, methodological heterogeneity, and variability in regenerative protocols may affect the overall certainty of the evidence [21,34]. Likewise, the limited number of currently available clinical trials continues to restrict the feasibility of performing robust subgroup analyses according to cell type, biomaterial characteristics, or periodontal defect morphology.

Among the principal strengths of the present review are the exclusive inclusion of controlled clinical trials, the application of random-effects models, and the performance of sensitivity analyses. This methodological approach allowed for interpretation of the findings from a more conservative and clinically responsible perspective, minimizing the risk of overestimating effects potentially influenced by the limited number of available studies.

Several limitations should be considered when interpreting the findings of the present review. First, substantial biological and clinical heterogeneity existed across studies because of differences in cellular sources, scaffolds, biomaterials, delivery strategies, and regenerative protocols, which may have contributed to variability in treatment effects. Second, comparator interventions ranged from conventional or cell-free approaches to active regenerative therapies such as GTR combined with Bio-Oss, potentially influencing the magnitude of the incremental benefit attributable to the cellular intervention. Third, important cell-related characteristics, including cell dose, viability, passage number, expansion protocols, and cellular characterization, were inconsistently reported, preventing meaningful subgroup analyses or meta-regression based on these variables. Fourth, the limited number of trials, small sample sizes, and inclusion of pilot, early-phase, and quasi-randomized studies contributed to imprecision and methodological uncertainty, resulting in low certainty of evidence across the evaluated outcomes. Fifth, although standardized mean differences allowed for quantitative synthesis across heterogeneous studies, their use reduced the direct clinical interpretability of the pooled effects compared with differences expressed in millimeters. Sixth, the unit of analysis varied across studies, with outcomes reported at the participant, tooth, or periodontal-defect level. Because the information required for additional clustering adjustments was not consistently available in the primary studies, residual within-participant correlation could not fully account for when multiple teeth or defects may have been contributed by the same participant. Seventh, the personalized-medicine perspective should be regarded as a framework for future research because the available trials lacked sufficient patient-level information and standardized reporting of factors such as smoking, systemic conditions, defect morphology, and cell-product characteristics to support formal patient-stratified analyses. Finally, although PRISMA-guided reporting, meta-analysis, and sensitivity analyses strengthen the transparency of the review, they cannot overcome limitations inherent to the primary studies; therefore, the present findings should be considered preliminary and hypothesis-generating, and adequately powered randomized clinical trials using standardized regenerative protocols are required before definitive conclusions can be established.

## 5. Conclusions

Cell-based regenerative therapies showed promising but inconclusive potential for periodontal regeneration. Small, non-significant effects were observed for clinical attachment level gain and probing depth reduction, whereas radiographic bone regeneration showed a larger favorable effect that remained statistically non-significant and was accompanied by substantial heterogeneity. Additional sensitivity analyses using mean differences and Hartung–Knapp adjustments did not materially change the overall interpretation, although uncertainty remained considerable, particularly for radiographic bone regeneration. The certainty of evidence was low across all evaluated outcomes.

The current evidence remains insufficient to support individualized clinical decision-making based on patient characteristics, periodontal defect features, cellular source, biomaterial selection, or delivery strategy. The variability observed across the available cellular products and regenerative protocols highlights the need for adequately powered, well-designed randomized clinical trials with standardized outcome assessment and more consistent reporting of patient, defect, and cell-product characteristics. Such evidence will be necessary before personalized periodontal regenerative strategies can be reliably incorporated into clinical practice.

## Figures and Tables

**Table 1:** Characteristics of included clinical studies evaluating mesenchymal stem cell-based therapies for periodontal regeneration.

Author (Year)	Country	Study Design	Participants	Total Number of Defects	Type of Control Condition	Defects in Controls	Type of MSC-Based Intervention	Defects with Stem Cells	Type of Other Regenerative Technique	Defects with Other Techniques	Periodontal Regeneration Outcomes
Liu et al. (2025) [22]	China	Multicenter report of two randomized, placebo-controlled clinical trials	132 patients overall; 94 patients in the post-hoc stage III subgroup used for quantitative synthesis	158 treated teeth overall; number of defects in the stage III subgroup not separately reported	Saline injection + scaling and root planing (SRP)	40 participants in the stage III post-hoc subgroup	Injectable allogeneic human dental pulp stem cells (DPSCs/hDP-MSCs)	54 participants in the stage III post-hoc subgroup	Scaling and root planing (SRP)	Applied to both intervention and control groups	Improvements in CAL, PD, and radiographic bone regeneration; quantitative data were derived from the post-hoc stage III subgroup
Sánchez et al. (2020) [23]	Spain	Quasi-randomized pilot clinical trial	19 patients	19 intrabony defects	Cell-free collagen scaffold	10	Autologous PDLSCs + collagen scaffold	9	Collagen scaffold alone	10	Moderate clinical improvement in CAL and PD without significant intergroup differences
Ferrarotti et al. (2018) [24]	Italy	Randomized clinical trial	29 patients	29 intrabony defects	Conventional regenerative periodontal surgery + biomaterial	14	DPSCs seeded on collagen scaffold	15	Conventional regenerative biomaterial	14	Greater CAL gain, PD reduction, and radiographic bone fill
Chen et al. (2016) [25]	China	Randomized clinical trial	41 patients	41 intrabony defects	GTR + Bio-Oss	21	Autologous PDLSCs + GTR + Bio-Oss	20	GTR + Bio-Oss without stem cells	21	Bone regeneration and clinical improvement with no significant differences between groups
Apatzidou et al. (2021) [26]	Germany	Phase I/IIa randomized clinical trial	26 patients	26 intrabony defects	Conventional flap surgery	8	Bone marrow-derived MSCs + scaffold	9	Scaffold without stem cells	9	Clinical improvements in CAL and PD with comparable outcomes among groups
Abdal-Wahab et al. (2020) [27]	Egypt	Randomized clinical trial	20 patients	20 intrabony defects	Conventional periodontal therapy	10	Gingival fibroblasts	10	Conventional periodontal treatment	10	Marked bone regeneration and clinical periodontal improvement

**Table 2:** Summary of findings and GRADE certainty of evidence for periodontal regeneration outcomes.

Outcome	Studies (*n*)	Participants (*n*)	Pooled Effect (95% CI)	Risk of Bias	Inconsistency	Indirectness	Imprecision	Publication Bias	Overall Certainty
Clinical attachment level (CAL) gain	5	199	SMD 0.17 (−0.12 to 0.45)	Serious ^1^	Not serious ^2^	Not serious	Serious ^3^	Could not be reliably assessed ^4^	Low ⨁⨁◯◯
Probing depth (PD) reduction	5	199	SMD 0.13 (−0.22 to 0.48)	Serious ^1^	Not serious ^5^	Not serious	Serious ^3^	Could not be reliably assessed ^4^	Low ⨁⨁◯◯
Radiographic bone regeneration	4	183	SMD 1.25 (−0.08 to 2.58)	Not serious ^6^	Serious ^7^	Not serious	Serious ^3^	Could not be reliably assessed ^4^	Low ⨁⨁◯◯

Abbreviations: CAL, clinical attachment level; CI, confidence interval; GRADE, Grading of Recommendations Assessment, Development and Evaluation; PD, probing depth; SMD, standardized mean difference. ^1^ Downgraded one level because the CAL and PD analyses included the quasi-randomized Sánchez et al. [23] trial, which was judged to have moderate overall risk of bias using ROBINS-I, and Apatzidou et al. presented some concerns in the RoB 2 assessment. ^2^ No serious inconsistency was identified for CAL because between-study heterogeneity was negligible (I^2^ = 0%). ^3^ Downgraded one level for imprecision because the 95% confidence interval crossed the null effect and the available sample size was limited. ^4^ Publication bias could not be reliably assessed because fewer than 10 studies contributed to each meta-analysis; no additional downgrading was applied. ^5^ No serious inconsistency was identified for PD (I^2^ = 33.0%). ^6^ No serious risk-of-bias concerns were identified among the randomized trials contributing to the radiographic bone regeneration meta-analysis. ^7^ Downgraded one level for serious inconsistency because substantial between-study heterogeneity was observed (I^2^ = 88.2%).

## Data Availability

The original contributions presented in this study are included in the article/Supplementary Materials. Further inquiries can be directed to the corresponding author.

## Supplementary Materials

The following supporting information can be downloaded at: https://www.mdpi.com/article/10.3390/jpm16090469/s1, Table S1. Complete database-specific search strategies, File S1: PRISMA checklist.
