# Supplementary material for: Cell-Based Therapies for Periodontal Regeneration: A Systematic Review and Meta-Analysis with Future Implications for Personalized Medicine
Source: J Pers Med. 2026 Sep 13;16(9):469. doi: 10.3390/jpm16090469 (PMC13608774; doi:10.3390/jpm16090469)
Supplement: Supplementary file 1 [file jpm-16-00469-s001.zip › Table S1. Complete database-specific search strategies.pdf]

Database	Search strategy	Records
**MEDLINE via PubMed**	(("Mesenchymal Stem Cells"[MeSH Terms] OR "mesenchymal stem cell*"[Title/Abstract] OR "mesenchymal stromal cell*"[Title/Abstract] OR "periodontal ligament stem cell*"[Title/Abstract] OR PDLSC*[Title/Abstract] OR "bone marrow stem cell*"[Title/Abstract] OR BMSC*[Title/Abstract] OR "dental pulp stem cell*"[Title/Abstract] OR DPSC*[Title/Abstract] OR "adipose-derived stem cell*"[Title/Abstract] OR ADSC*[Title/Abstract] OR "gingival mesenchymal stem cell*"[Title/Abstract] OR "stem cell therap*"[Title/Abstract]) AND ("Periodontitis"[MeSH Terms] OR "Periodontal Regeneration"[MeSH Terms] OR periodontitis[Title/Abstract] OR "periodontal regeneration"[Title/Abstract] OR "periodontal defect*"[Title/Abstract] OR "intrabony defect*"[Title/Abstract] OR "intra-bony defect*"[Title/Abstract]))	**1023**

**Embase**	('mesenchymal stem cell'/exp OR 'mesenchymal stem cell*':ti,ab,kw OR 'mesenchymal stromal cell*':ti,ab,kw OR 'periodontal ligament stem cell*':ti,ab,kw OR PDLSC*:ti,ab,kw OR 'bone marrow stem cell*':ti,ab,kw OR BMSC*:ti,ab,kw OR 'dental pulp stem cell*':ti,ab,kw OR DPSC*:ti,ab,kw OR 'adipose derived stem cell*':ti,ab,kw OR ADSC*:ti,ab,kw OR 'gingival mesenchymal stem cell*':ti,ab,kw OR 'stem cell therap*':ti,ab,kw) AND ('periodontal regeneration'/exp OR 'periodontitis'/exp OR 'periodontal attachment loss'/exp OR 'alveolar bone loss'/exp OR 'periodontal regeneration':ti,ab,kw OR periodontitis:ti,ab,kw OR 'periodontal attachment loss':ti,ab,kw OR 'alveolar bone loss':ti,ab,kw OR 'periodontal defect*':ti,ab,kw OR 'intrabony defect*':ti,ab,kw OR 'bone regeneration':ti,ab,kw)	**75**

**Web of Science Core Collection**	TS=(("mesenchymal stem cell*" OR "mesenchymal stromal cell*" OR "periodontal ligament stem cell*" OR PDLSC* OR "bone marrow stem cell*" OR BMSC* OR "dental pulp stem cell*" OR DPSC* OR "adipose-derived stem cell*" OR ADSC* OR "gingival mesenchymal stem cell*" OR "stem cell therap*") AND ("periodontal regeneration" OR periodontitis OR "periodontal attachment loss" OR "alveolar bone loss" OR "guided tissue regeneration" OR "periodontal defect*" OR "intrabony defect*" OR "bone regeneration"))	**220**

**Cochrane Library / Cochrane Oral Health Group Trials Register**	(("mesenchymal stem cell*" OR "mesenchymal stromal cell*" OR "periodontal ligament stem cell*" OR PDLSC* OR "bone marrow stem cell*" OR BMSC* OR "dental pulp stem cell*" OR DPSC* OR "adipose-derived stem cell*" OR ADSC* OR "gingival mesenchymal stem cell*" OR "stem cell therap*"):ti,ab,kw) AND (("periodontal regeneration" OR periodontitis OR "periodontal attachment loss" OR "alveolar bone loss" OR "periodontal defect*" OR "intrabony defect*" OR "bone regeneration"):ti,ab,kw)	**40**
**Total database records**		**1358**
